# Proteome expression profiling of red blood cells during the tumorigenesis of hepatocellular carcinoma

**DOI:** 10.1371/journal.pone.0276904

**Published:** 2022-11-08

**Authors:** Shufang Wang, Guibin Wang, Shichun Lu, Jiaying Zhang, Wenwen Zhang, Yuanyuan Han, Xiaoyu Cai, Yuan Zhuang, Fei Pu, Xin Yan, Zhiwei Tu, Liang Wang, Xirui Huang, Bin Fan, Deqing Wang, Zhaojun Zhang

**Affiliations:** 1 The Blood Transfusion Department, First Medical Center of Chinese PLA General Hospital, Beijing, China; 2 State Key Laboratory of Proteomics, Beijing Proteome Research Center, National Center for Protein Sciences (Beijing), Beijing Institute of Lifeomics, Beijing, China; 3 Faculty of Hepato-Pancreato-Biliary Surgery, Chinese PLA General Hospital, Beijing, China; 4 Beijing Institute of Genomics & China National Center for Bioinformation, Institute for Stem Cell and Regeneration, Chinese Academy of Sciences, Beijing, China; 5 School of Medicine, Guizhou University, Guiyang, China; 6 Xiongxian County Center for Disease Control and Prevention, Baoding, China; 7 Department of Pathology, The First Medical Center of Chinese PLA General Hospital, Beijing, China; 8 University of Chinese Academy of Sciences, Beijing, China; 9 Beijing Key Laboratory of Genome and Precision Medicine Technologies, Beijing, China; Pacific Northwest National Laboratory, UNITED STATES

## Abstract

The early diagnosis of hepatocellular carcinoma (HCC) has not been clinically elucidated, leading to an increased mortality rate in patients with HCC. HCC is a systemic disease related to disorders of blood homeostasis, and the association between red blood cells (RBCs) and HCC tumorigenesis remains elusive. We performed data-independent acquisition proteomic analyses of 72 clinical RBC samples, including HCC (n = 30), liver cirrhosis (LC, n = 17), and healthy controls (n = 25), and characterized the clinical relevance of RBCs and tumorigenesis in HCC. We observed dynamic changes in RBCs during HCC tumorigenesis, and our findings indicate that, based on the protein expression profiles of RBCs, LC is a developmental stage closely approaching HCC. The expression of hemoglobin (HbA and HbF) in peripheral blood dynamically changed during HCC tumorigenesis, suggesting that immature erythroid cells exist in peripheral blood of HCC patients and that erythropoiesis is influenced by the onset of LC. We also identified the disrupted autophagy pathway in RBCs at the onset of LC, which persisted during HCC tumorigenesis. The oxytocin and GnRH pathways were disrupted and first identified during the development of LC into HCC. Significantly differentially expressed SMIM1, ANXA7, HBA1, and HBE1 during tumorigenesis were verified as promising biomarkers for the early diagnosis of HCC using parallel reaction monitoring technology. This study may enhance the understanding of HCC tumorigenesis from a different point of view and aid the early diagnosis of HCC.

## Introduction

Liver cancer is the third leading cause of cancer-related deaths globally, and hepatocellular carcinoma (HCC) is the most common primary liver cancer in adults. HCC is difficult to treat and is easily misdiagnosed at an early stage. α-fetoprotein (AFP) is the most widely used biomarker in the clinical diagnosis of HCC with a sensitivity of ~39–65% [1]. However, most patients with HCC have reached the middle or late stage, and less than 20% of patients qualify for surgical resection [2, 3]. A novel strategy for the early diagnosis of HCC is necessary to improve the clinical treatment of patients with HCC.

Liver cirrhosis (LC) is a common developmental stage of hepatitis B to HCC. Hepatitis B patients develop LC within ~5–10 years without suitable antiviral treatment accompanied by an unhealthy lifestyle due to abnormal liver function. Clinically, ~10% of patients with LC further develop HCC within five years. LC is a crucial stage for the early diagnosis of HCC.

The liver is the main organ of fetal erythropoiesis and the site for erythropoiesis in adults in certain disorders [4]. Erythropoiesis-associated disorders occur in patients with HCC. For example, erythrocytosis is one of the paraneoplastic syndromes in HCC, accounting for 3–12% of HCC cases that show an increase in the concentration of RBCs and hemoglobin [5], in which HCC cells are responsible for the production of erythropoietin for erythrocytosis [6]. Increased RBC distribution width, a main feature of RBCs, is also clinically observed in liver disease patients [7]. Moreover, erythroblast-like Ter-cells are observed in enlarged-spleen patients with HCC, and the elevated artemin in serum secreted from these cells highly correlates with HCC progression [8]. As a systemic disease related to disorders of blood homeostasis, cancer causes detectable changes in gene expression in blood cells and plasma [9–11]. Cancer-educated platelets, erythrocytes, or leukocytes in blood have potential applications in cancer diagnosis [12–14]. Therefore, we speculate that HCC tumorigenesis is associated with the molecular characteristics of RBCs.

The rapidly expanding field of HCC-biomarker identification provides a fast-growing list of biomarker candidates, including microRNAs (miRNAs) in plasma [15], epigenetic 5-hydroxymethyl cytosine in peripheral blood [16], and proteins in serum [17]. However, the molecular characteristics of RBCs have rarely been explored to identify biomarkers for the early diagnosis of HCC.

In this study, we revealed the association of RBCs with HCC tumorigenesis by exploring protein profiles of RBCs in a cohort of subjects, including patients with LC, HCC, and healthy individuals. We characterized molecular alterations in RBCs that are closely associated with HCC tumorigenesis, including erythroid-specific globins and unique proteins and signaling pathways. Novel biomarkers in RBCs for the early diagnosis of HCC were discovered, including small integral membrane protein 1 (SMIM1) and annexin A7 (ANXA7). This study is the first to establish the link between HCC tumorigenesis and proteins in RBCs that provides a novel strategy for the early diagnosis of HCC. Our study may improve translational research and application in the diagnosis of HCC.

## Materials and methods

### Patients

To investigate whether protein molecular characteristics are associated with the tumorigenesis of HCC, 30 HCC patients with HBV infection, 17 LC patients without HCC, and 25 healthy individuals were enrolled in this study. All patient diagnoses were confirmed by pathological examination. The age of the cohort ranged from 40 to 60 years. The selected patients had no history of blood transfusions or gastrointestinal bleeding. Patients with LC or HCC were treated for the first time at our hospital, and blood samples were collected before surgery. Clinical data for each subject concerning AFP, alanine transaminase (ALT), aspartate aminotransferase (AST), hemoglobin, and blood cells were collected from medical records. We collected 2 mL of peripheral blood from each patient who underwent clinical examination from March 2020 to June 2020 and conducted the proteome- and validation-related assays during the same period. We performed statistical analyses of clinical data across the cohort of subjects. Written informed consent was obtained from all subjects. This study was performed following ethical standards of the institutional research committee and the latest Declaration of Helsinki and was approved by the ethics committee of the PLA General Hospital.

### Isolation of red blood cells

To investigate RBC protein alterations during the tumorigenesis of HCC by data-independent acquisition (DIA) mass spectrometry, 2 mL of peripheral blood from each subject was collected in EDTA anticoagulant tubes. We diluted fresh blood with 1× PBS in the ratio of 1:1. After the lymphocyte separation solution was added to 15 mL centrifuge tube, we added the diluted blood into the separation solution (1:1), centrifuged at 2600 rpm for 20 min at room temperature, and then, the top layer of plasma and the middle layer of platelets and white blood cells were removed to obtain the bottom layer of RBCs. Next, 5 mL of normal saline was added to mix and wash the cells. After three rounds of centrifugation at 3000 rpm for 5 min, RBCs were collected and frozen at -80°C for later use.

### Liquid chromatography–tandem mass spectrometry analysis

A total of 72 individual RBC samples were analyzed by DIA mass spectrometry. One microliter of RBC sample for each subject was diluted with 50 mM NH_4_HCO_3_, and the proteins were reduced using 10 mM dithiothreitol at 56°C and alkylated using 50 mM iodoacetamide in the dark. Protein digestion was performed using the filtered-aided sample preparation method for mass spectrometry. The concentration was measured using NanoDrop One (ThermoFisher Scientific). A spectral library was generated using the data-dependent acquisition (DDA) method, as previously reported [18]. Ten micrograms of peptides from each sample were pooled and separated into 10 components by high pH reversed-phase chromatography. Two micrograms of peptides per component were separated using a C18 analysis column (150 μm × 15 cm, 1.9 μm) with the Easy NanoLC 1200 system (ThermoFisher Scientific) at a 75 min gradient and analyzed by Q Exactive HF mass spectrometry (ThermoFisher Scientific). The gradient, with a flow rate of 600 nL/min, was set as follows: 10–14% solvent B for 12 min, 14–26% solvent B for 45 min, and 26–42% solvent B for 10 min. For DDA analysis, the electrospray voltage was set at 2.1 kV. The full MS1 scan ranged from 300 to 1400 *m*/*z* at a resolution of 60 k. Twenty MS/MS spectrums were scanned per cycle at a resolution of 15 000. MS1 and MS2 AGC targets were set at 3e^6^ and 5e^4^ corresponding to maximum injection times of 80 ms and 40 ms, respectively. The isolation window was set at 1.6 Th, and dynamic exclusion was set at 18 s. For DIA analysis, the MS1 ranged from 350 to 1400 *m*/*z* at a resolution of 60 k. A total of 30 windows covering 400–1250 *m*/*z* were used for DIA with a resolution of 30 k and 3e^6^ AGC.

### Bioinformatics analysis

Hierarchical clustering was performed using in-house R-scripts. An unpaired Wilcoxon rank-sum test was used to identify differentially expressed proteins (DEPs). Proteins with log_2_-fold changes > 0.58 or < -0.58 and with *P*-values < 0.05 were identified as DEPs. Functional enrichment was performed using gene ontology (GO), Kyoto Encyclopaedia of Gene and Genomes (KEGG), and STRING databases. An adjusted *P*-value threshold cut-off was set at 0.05. Diagrams are shown with significance (−log_2_ transformed) and protein number identified in the relevant protein sets.

### Flow cytometry

To analyze changes in hemoglobin expression during HCC tumorigenesis, 2 mL of peripheral blood from another batch of subjects including eight HCC, nine LC patients, and five healthy individuals were analyzed. Samples were fixed in a 0.05% glutaraldehyde solution in 1 × PBS for 10 min, permeabilized for 5 min in 0.01% Triton X-100 in 1 × PBS or 1% FBS for 5 min, and stained with PE-conjugated fetal hemoglobin antibody (Miltenyi Biotec) and AF647-conjugated hemoglobin-β antibody (Santa Cruz Biotechnology) for 15 min in the dark. After incubation, cells were washed twice and resuspended in 200 μL 1 × PBS buffer to prepare the cell suspension. A BD FACSAria II instrument was used for flow cytometric analysis, and the data analysis was performed using FlowJo software (version 7.6, Tree Star).

### Parallel reaction monitoring validation

The validation of proteins was carried out by parallel reaction monitoring (PRM) technology [19]. Briefly, PRM analyses were performed using a 40 min gradient LC with a flow rate of 600 nL/min: 8–13% solvent B for 2 min, 13–35% solvent B for 24 min, and 35–45% solvent B for 4 min. The mass spectrometry data were acquired using the following parameters: PRM scans were performed at a resolution of 30 000 at 200 *m*/*z* with an individual isolated window of 1.6 Th, and the retention time window was set at ± 4 min, using a maximum injection time of 40 ms. The normalized collision energy of 27 in an HCD collision cell was employed for fragmentation. PRM data analysis and data integration were performed using Skyline software (version 3.5.0).

### Immunohistochemistry

Paraffin-embedded tissue sections of HCC and matched controls were analyzed by immunostaining. Anti-SMIM1 (Cusabio Technology) was used at a dilution of 1:100. Digital imaging was performed using LAS version 4.5 (Leica DM 2000). Images were acquired using the HistoFAXS system, and the staining was visualized using K-viewer software.

## Results

### Clinical characteristics of RBCs associated with HCC tumorigenesis

**Fig 1:**
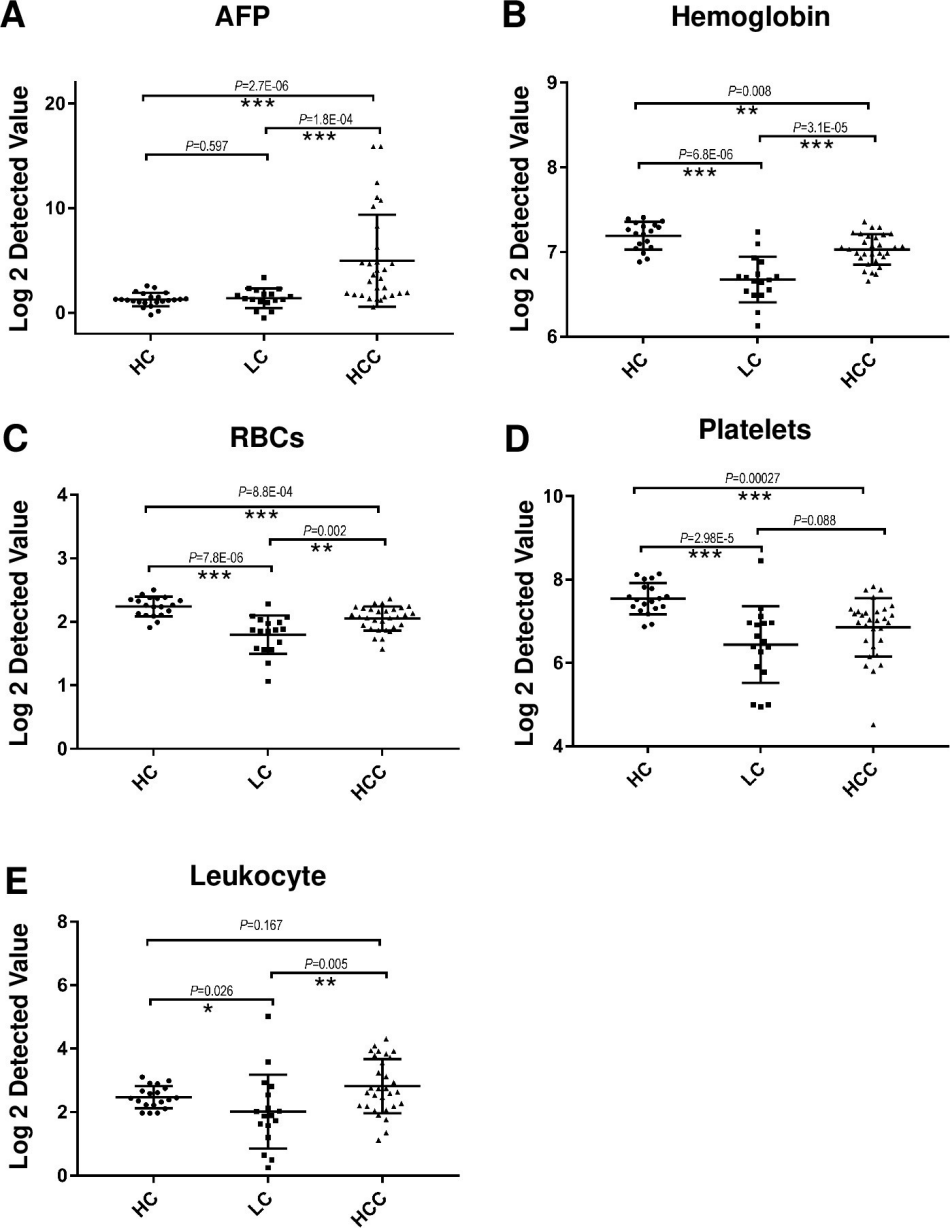
Clinical characteristics of peripheral blood during HCC tumorigenesis. A, B. Changes in peripheral blood AFP and hemoglobin levels in a cohort of patients with LC, HCC, and HC during HCC tumorigenesis. AFP was clinically detected by electrochemiluminescence immunoassays. Hemoglobin was clinically detected by the cyanmethemoglobin method. C–E. Changes in the number of peripheral blood RBCs, platelets, and leukocytes in a cohort of patients with LC, HCC, and HC. Blood cells were clinically detected using a fully automatic hematology analyzer. Statistical results were analyzed using the Wilcoxon rank-sum test. ***, *P* < 0.001; **, *P* < 0.01, *, *P* < 0.05.

To explore the relationship between the tumorigenesis of HCC and RBCs, we collected clinicopathologic characteristics of LC (n = 17), HCC (n = 30), and healthy controls (HC, n = 25) (S1 Table). The detected ALT, AST, and AFP levels in peripheral blood were higher in HCC than those in LC and HC, indicating functional damage of the liver at the onset of HCC (S1 Table). The AFP level in HCC fluctuated extensively (Fig 1A), and more than 50% of patients with HCC fell within the normal range, which may have resulted in the missed detection of HCC cases. The AFP level in LC is similar to that in HC, indicating that AFP can not be used as a biomarker for the early diagnosis of HCC. The level of hemoglobin (Fig 1B) and RBCs (Fig 1C) in peripheral blood were significantly lower in LC than in HC, suggesting that RBCs are influenced in LC. Interestingly, this disruption gradually recovered in established HCC, approaching HC, indicating the association of molecular characteristics in RBCs with HCC tumorigenesis. Tumor-educated blood platelets were previously characterized to distinguish six types of cancers besides HCC from healthy controls using mRNA profiling [14]. We first observed that platelets were also associated with HCC tumorigenesis, as platelet levels in peripheral blood changed in a trend similar to that in RBCs (Fig 1D). Leukocyte levels changed slightly during HCC tumorigenesis. Taken together, our results demonstrate that the changes in RBCs during tumorigenesis may reflect the progression of HCC.

### Proteomic profiles of RBCs in LC and HCC patients differ from those of HC

**Fig 2:**
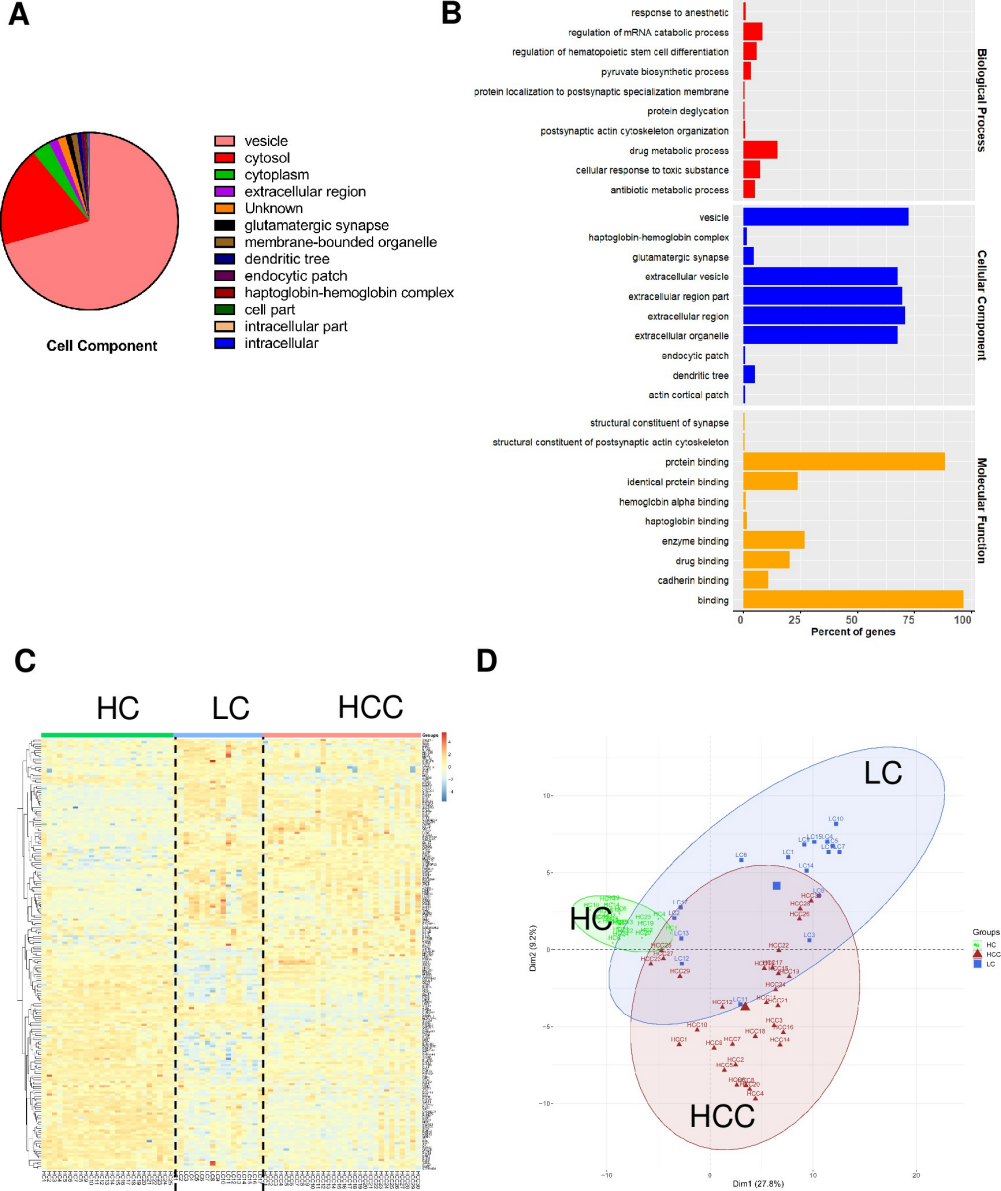
Analysis of identified RBC proteins in a cohort of patients with HCC, LC, and HC. A. Cellular localization of 659 identified RBC proteins by GO analysis. B. Functional enrichment of identified RBC proteins by GO analysis. C. Hierarchical clustering analysis of LC, HCC, and HC groups based on 659 DEPs. D. PCA analysis of LC, HCC, and HC groups based on DEPs.

RBCs clinically indicated the progression of HCC. However, the molecular characteristics of RBCs during HCC tumorigenesis have not been investigated. Here, we utilized DIA mass spectrometry to analyze protein profiles in RBCs from the cohort, including HCC (n = 30), LC (n = 17), and HC patients (n = 25) (S1 Table). A total of 659 proteins were identified for characterization (S2 Table), of which most proteins are present in vesicles and cytosols of RBCs (Fig 2A) and perform a variety of cellular responses and metabolism-related functions (Fig 2B). The hierarchical clustering analysis showed that protein expression profiles of RBCs in LC and HCC patients differed from those of HC, and LC is closer to HCC (Fig 2C), which was also revealed by PCA analysis (Fig 2D). In terms of the RBC proteome, we demonstrated that the pathology of LC is a developmental stage towards HCC and that LC is a crucial stage of HCC development. Dynamic changes of proteins in LC may facilitate the early diagnosis of HCC.

### Erythroid-specific proteins may indicate the pathological process of HCC

**Fig 3:**
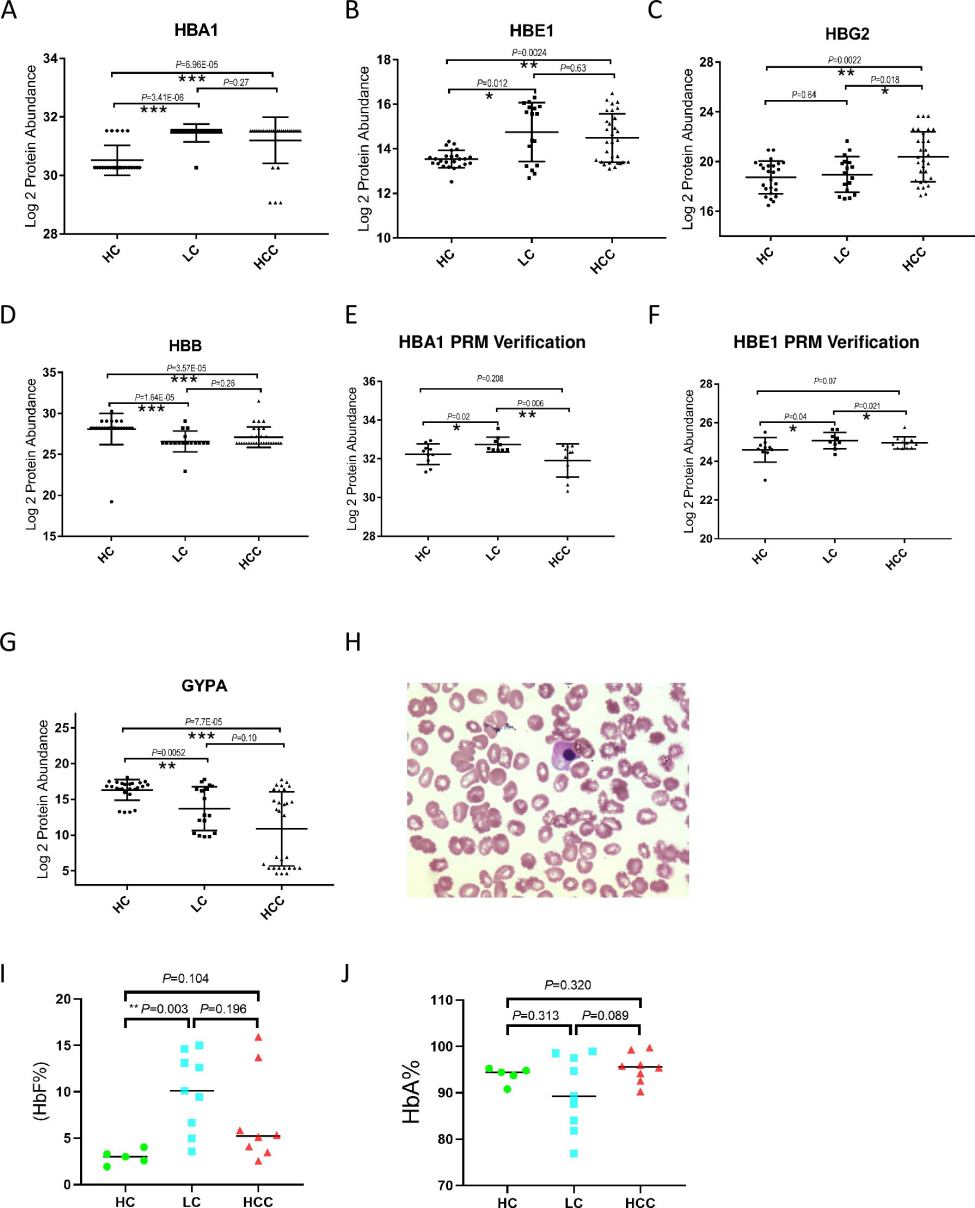
Analysis of erythroid-specific protein expression in a cohort of patients with HCC, LC, and HC. A–D. Analysis of globins expression changes in the cohort of patients with LC (n = 17), HCC (n = 30), and HC (n = 25). The expression was derived from DIA mass spectrometry data. E–F. The expression of HBA1 and HBE1 in RBCs during tumorigenesis was verified using the PRM strategy in another batch of clinical samples from LC (n = 9), HCC (n = 11), and HC (n = 10). G. Analysis of GYPA expression changes in the cohort of patients with LC (n = 17), HCC (n = 30), and HC (n = 25). H. Nucleated erythroid cells from HCC patients were observed under a microscope. I–J. Statistics of RBCs respectively expressing HbF (I) and HbA (J) in peripheral blood samples from HCC (n = 8), LC (n = 9), and HC (n = 7). The cell number was calculated by flow cytometry analysis. Statistical results were analyzed using the Wilcoxon rank-sum test. ***, *P* < 0.001; **, *P* < 0.01, *, *P* < 0.05.

The clinical characteristics of RBCs and hemoglobin in peripheral blood from the cohort fluctuated during HCC tumorigenesis. We next investigated the relationship between expression profiles of erythroid-specific proteins and the progression of HCC, which possibly indicates biomarkers for the early diagnosis of HCC. We observed that the components of hemoglobin, including HBA1, HBE1, HBG2, and HBB, showed differentiated expression during the tumorigenesis of HCC (Fig 3A–3D). Specifically, HBA1 expression increased significantly during LC, while HBB expression decreased in the same stage. HBG2, which is the predominant fetal globin at birth, remained at a similar expression level as HC during LC. However, HBG2 levels were the only globin levels that differed in the HCC group compared with those in the LC group, suggesting that HBG2 possibly indicates the progression of LC into HCC. As an embryonic globin, HBE1 expression increases during both LC and HCC.

PRM is a targeted proteomics technology based on high-resolution and high-precision mass spectrometry, which selectively detects target proteins and peptides to achieve absolute quantification of the target protein or peptide. In this study, we verified the expression of differentially expressed globins in another cohort of LC, HCC, and HC using RPM technology (S3 and S4 Tables) and observed that the expression of HBA1 and HBE1 in LC is higher than that in HC. However, the expression significantly decreased during the development of LC into HCC (Fig 3E and 3F). This pattern differed from that of proteomic data.

Consistently, we observed the decreased expression of GYPA, a specific marker of mature erythrocytes, in RBCs of LC and HCC patients compared with those of HC; however, no difference was observed in the RBCs of LC and HCC patients (Fig 3G). Nucleated erythroid cells are usually present in peripheral blood in LC [20, 21]. We also observed nucleated erythroid cells in the peripheral blood of patients with HCC (Fig 3H). These results indicate that immature erythroid cells were present in peripheral blood of patients with HCC. To test this hypothesis, we ennumerated erythroid cells respectively expressing HbF and HbA in peripheral blood from another cohort of HCC and LC patients and HC using flow cytometry analysis. Interestingly, we observed that the number of erythroid cells expressing HbF is significantly higher in LC patients than in HC patients (Fig 3I), indicating that the production of erythroid cells is initially affected during LC and that immature erythroid cells are released into peripheral blood at this stage. Consistently, we observed the lowest number of erythroid cells expressing HbA consisting of two α-chains together with two β-chains in LC compared with that in HC and HCC (Fig 3J), even though no statistically significant difference was observed. Our results demonstrated that changes in the expression of erythroid-specific proteins during HCC tumorigenesis indicated pathological progression and that the increase of HbF in LC may be used for the early diagnosis of HCC.

### Impairments in RBCs at the onset of liver cirrhosis

**Fig 4:**
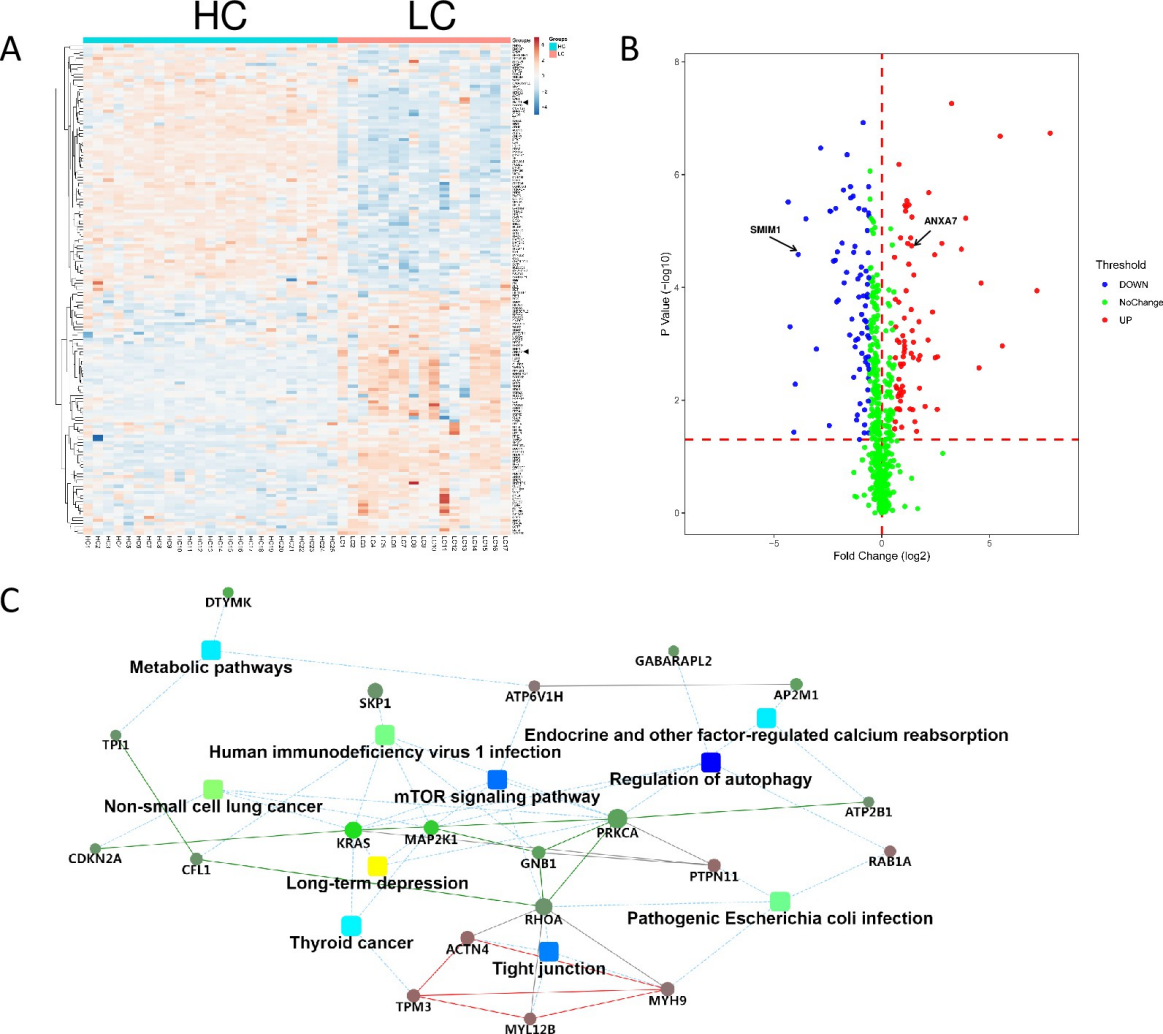
Alterations in RBCs at the onset of LC. A. Hierarchical clustering analysis of LC and HC groups based on DEPs. A solid triangle indicates SMIM1 and ANXA7, respectively. B. Volcanic plot of DEPs from protein differential expression analysis in LC and HC groups (*P* < 0.05). SMIM1 and ANXA7 are highlighted with an arrow. C. Interactive networks that connect disrupted pathways and proteins in RBCs at the onset of LC. The networks were constructed using significant DEPs in LC and HC groups according to STRING analysis.

We next explored the disturbance of RBCs at the onset of LC, which might be a sign of the development of HCC. A total of 157 DEPs (79 upregulated and 78 downregulated) was identified in RBCs at the onset of LC (S5 Table and Fig 4A and 4B). More proteins were altered than those in the progression of LC to HCC (57 DEPs), indicating that the onset of LC leads to drastic changes in RBCs during the tumorigenesis of HCC.

Autophagy is a major role player in LC and is considered an anti-fibrosis pathway, providing survival signals for hepatocytes and acting as the gatekeeper of HCC [22]. We also observed the significantly disrupted autophagy pathway at the onset of LC in RBCs. The impairment of mTOR and tight junction pathways was firstly characterized in LC (Fig 4C).

Interestingly, we observed two proteins, SMIM1 and ANXA7, that were associated with the cellular characteristics of RBCs [23–25] and the tumorigenesis of HCC and functions of erythroid cells, respectively [26–29]. Since the expression of these two proteins are significantly altered in RBCs at the onset of LC (Fig 4A and 4B), we speculated that they are potential biomarkers for the early diagnosis of HCC. The differential expression of these two proteins were further verified with clinical samples using RPM technology. In summary, these disrupted proteins or pathways in RBCs at the onset of LC may facilitate the early diagnosis of HCC.

### Changes in RBCs from LC to HCC during tumorigenesis

**Fig 5:**
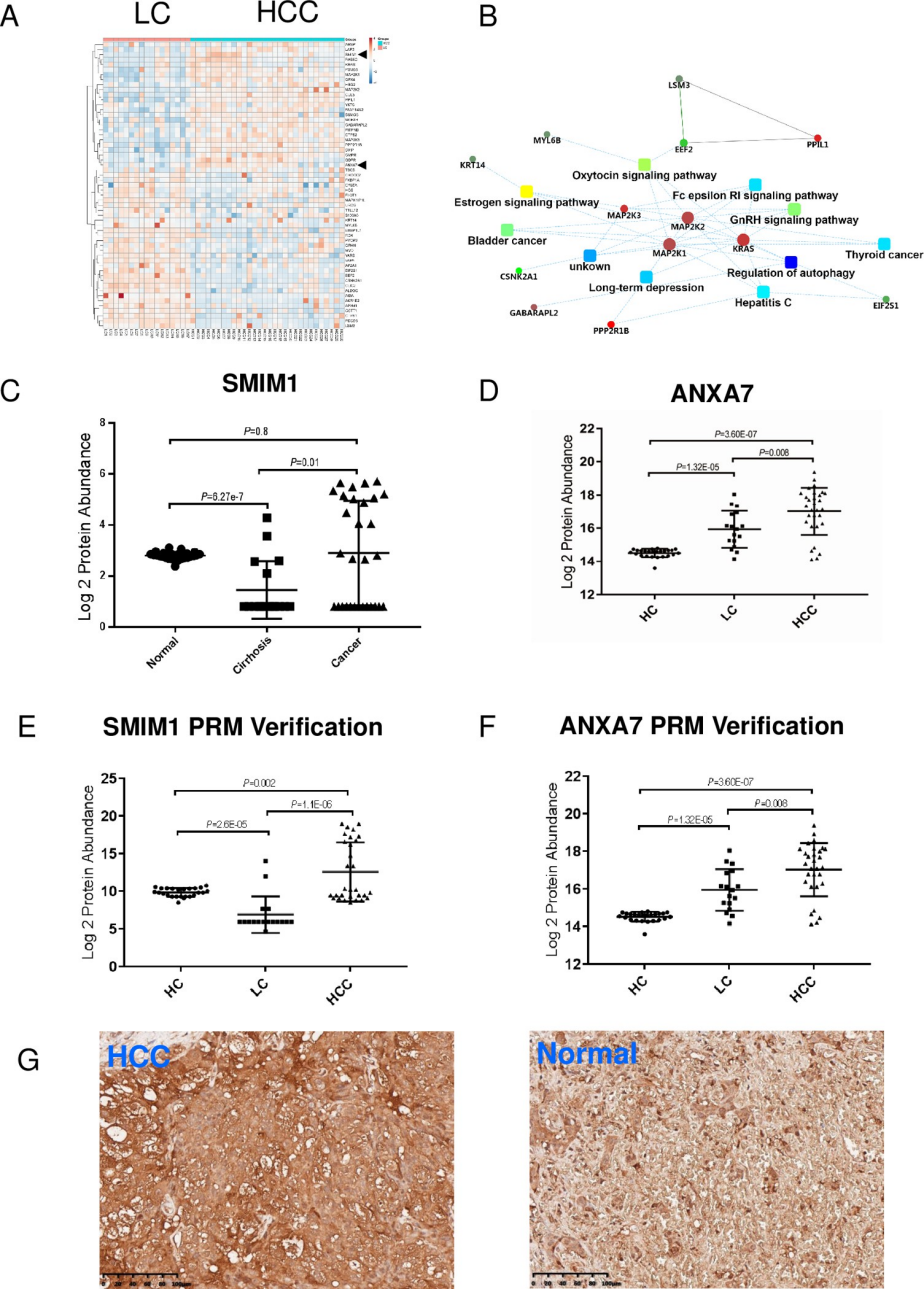
Alterations in RBCs and potential biomarker identification during tumorigenesis of LC to HCC. A. Hierarchical clustering analysis of HCC and LC groups with DEPs. B. Interactive networks regulating the tumorigenesis of LC to HC. Networks connect the disrupted pathways and proteins in RBCs and were constructed using DEPs in LC and HCC groups. C–D. Analysis of SMIM1 and ANXA7 expression in a cohort of patients with LC, HCC, and HC, respectively. The protein expression was calculated from mass spectrometry data and a significant difference analysis was conducted between groups. E–F. The expression trends of SMIM1 and ANXA7 during tumorigenesis were verified using a PRM strategy with another batch of clinical samples from LC (n = 9), HCC (n = 11), and HC (n = 10). G. Immunostaining of SMIM1 in normal liver and HCC tissue.

Liver cirrhosis is a developmental stage that progresses into HCC, during which the maturation of RBCs in peripheral blood is influenced. We next explored the changes in RBCs during LC–HCC transition by analyzing DEPs in LC and HCC patients. We identified 57 DEPs (26 upregulated and 31 downregulated) in HCC compared with LC (S6 Table and Fig 5A), and these disturbed DEPs were also enriched in the autophagy pathway (Fig 5B) that is necessary for the suppression of spontaneous tumorigenesis via a cell-intrinsic mechanism. In addition, the impairment of autophagy initiates spontaneous liver tumorigenesis in aged mice [30, 31]. This finding suggested the disruption of the gatekeeper of HCC during this process. The estrogen pathway (Fig 5B), another HCC-associated pathway, is also disrupted during this process [32]. However, the disrupted oxytocin and gonadotropin-releasing hormone (GnRH) pathways were first identified during this process.

During this transition, we observed that SMIM1 expression is downregulated at the onset of LC and is gradually increased towards a normal expression level in HCC (Fig 5C), and ANXA7 expression gradually increased during HCC tumorigenesis (Fig 5D). The PRM assay confirmed the dynamic changes in SMIM1 and ANXA7 expression levels in RBCs during HCC tumorigenesis using clinical samples (Fig 4E and 4F and S3 and S4 Tables). SMIM1 expression dramatically decreased in LC and gradually increased in HCC, while ANXA7 expression continuously increased from HC to HCC, both of which may indicate the tumorigenesis of HCC. The dramatic decrease of SMIM1 expression in RBCs from LC patients may act as an early diagnostic biomarker for HCC. Moreover, we tested SMIM1 expression levels in HCC tissue. The results showed that SMIM1 is expressed in both HCC tissue and precancerous lesions (Fig 5G) but at higher levels in HCC. The underlying mechanism of the association of SMIM1 expression with HCC progression and the production of erythroid cells needs further exploration.

### Pathways involving erythropoiesis are affected in HCC and HC

**Fig 6:**
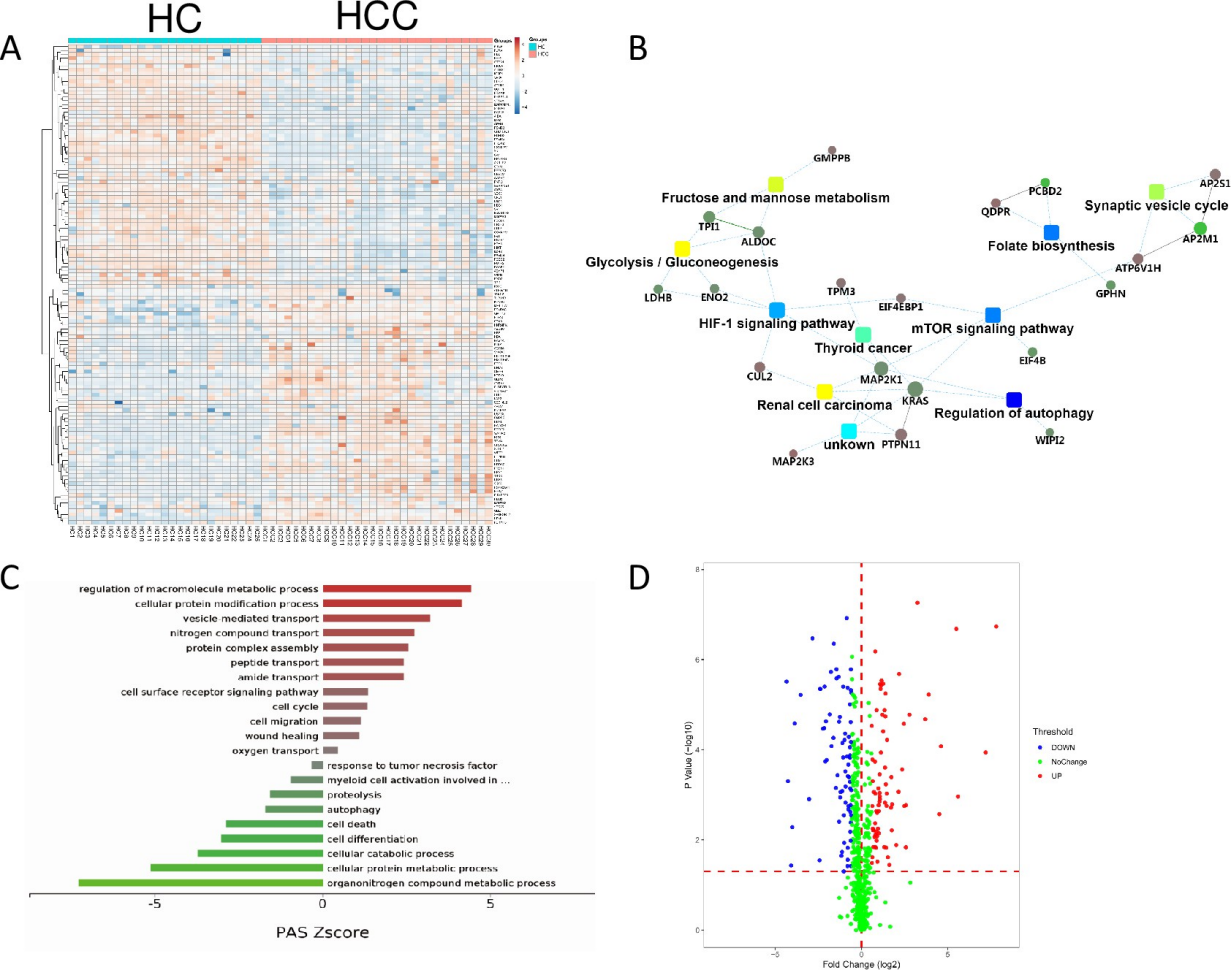
Erythropoiesis-related pathways are affected in HCC and HC RBCs. A. Hierarchical clustering analysis of HCC and HC groups using DEPs. B. Interactive networks involving disrupted pathways and proteins constructed using DEPs in HCC and HC groups. C. Enriched biological processes with the highest PAS Z score. D. Volcanic plot of DEPs in HCC and HC groups (*P* < 0.05).

We next investigated the changes in RBCs that could be caused by HCC tumorigenesis in HCC patients by comparing them with those in HC. The DEPs in HC and HCC patients clearly distinguished these two stages (Fig 6A). The disruption of oxygen transport, the folate metabolic pathway, HIF-1 pathway, and glycolysis pathway are closely related to erythroid differentiation [33–36], suggesting that erythropoiesis is abnormal in established HCC (Fig 6B and S7 Table). Interestingly, the disrupted mTOR pathway, first identified in LC in this study, was also identified in established HCC (Fig 6B). A few cancer-related pathways were also altered in HCC, including autophagy, cell death, protein degradation, proteolysis, response to tumor necrosis, and a variety of metabolic pathways that are enriched by the downregulated proteins (Fig 6C), demonstrating that cancer-associated dysfunctions are also present in RBCs in established HCC.

## Discussion

HCC is rarely detected early and is usually fatal within a few months of diagnosis. The early diagnosis of HCC is currently the most important step in HCC management. Increasing attention has been paid to blood testing as a non-invasive screening for tumors. In addition to circulating tumor cells in peripheral blood, some factors released by tumor cells are found in plasma, and a large number of tumor biomarkers is characterized, including CA125 and AFP, of which CA125 is also a sensitive factor in LC [37, 38]. Transcriptional information carried by platelets in peripheral blood can be utilized to estimate the onset and type of tumors [14]. RBCs have a life span of 120 days, which may change during the circulation of peripheral blood undergoing tumorigenesis. We hypothesize that RBCs enable clinical advances in blood-based “liquid biopsies” and that certain biochemical properties in RBCs are biomarkers for cancer diagnosis, even at earlier stages.

Hepatitis B patients may gradually develop liver cancer. Therefore, the analysis of the transformation of LC into HCC is indispensable for early diagnosis. We found that the routine blood indices of LC patients were abnormal and that the protein expression profile of RBCs was significantly different from that of healthy individuals. The number of RBCs and PLTs in LC patients is less than that in healthy individuals, and the number of RBCs in patients with LC is also less than that in patients with HCC, but the number of PLTs is not less than that in patients with HCC. We speculate that disrupted protein levels are biomarkers for the progression of LC to HCC. SMIM1, a small and conserved membrane protein, is an antigen of the Vel blood group and participates in red blood cell formation [23, 24]. SMIM1 expression decreases during LC but increases 18-fold in HCC, returning to near normal levels (Fig 5C and 5E), suggesting that SMIM1 may indicate the development of LC in HCC. The dramatic decrease of SMIM1 expression in LC may be an early sign of HCC. We suggest that the combined detection of SMIM1, AFP, and CA125 expression improves the accuracy of the early diagnosis of HCC. ANXA7, a Ca^2+^-binding protein that is involved in membrane organization and dynamics, plays a role in promoting the proliferation of liver cancer, and a loss of function decelerates the proliferation of liver cancer [39]. We found that ANXA7 expression may reflect the development of HCC since its expression gradually increases during tumorigenesis. ANXA7 may be a potential biomarker for the early diagnosis of HCC.

In this study, we first observed the alteration in globin expression levels in RBCs during tumorigenesis. Globins, including myoglobin, hemoglobin, cytoglobin, and neuroglobin, are present in all kingdoms of living organisms where they display a variety of functions, including O_2_ sensing, transport, storage, and heme-based catalysis [40]. Myoglobin and cytoglobin are tumor suppressors in breast and lung tumors. The loss of cytoglobin accelerates liver fibrosis and cancer development despite its etiology in mouse models of chronic liver injury [41]. Neuroglobin is a unique globin identified as a critical player in cancer cell adaptation and resistance to detrimental oxidative stress conditions [42]. β-globin expression is selectively increased in cancer cells, mediating a cytoprotective effect during blood-borne metastasis [43]. The normal adult hemoglobin is a predictive clinical indicator for liver cancer combined with CA-125 [38]. Hemoglobin is the most abundant protein in RBCs. Two α-chains together with two γ-chains constitute fetal hemoglobin (HbF: α2γ2), which is normally replaced by adult hemoglobin (HbA: α2β2) at birth. In this study, we observed that the expression of hemoglobin, especially HbF, dramatically changed during the tumorigenesis of HCC. The maturity of erythroid cells in HCC patients decreases. Consistently, embryonic and fetal globin expression are increased in established HCC, suggesting that immature erythroid cells are present under HCC conditions and that γ- and/or ε-chain production continues into adulthood. The alteration of erythroid-specific globin expression may indicate the progression of HCC and may serve as a biomarker for the early diagnosis of HCC. This finding may constitute an advancement in clinical application and translational research in this field, even though the molecular mechanism underlying the association of the biosynthesis of different types of erythroid-specific globins in peripheral blood and tumorigenesis of HCC remains unexplored.

Interestingly, we observed that the preoperative anemia rate in HCC (34.45%) is lower than that in many cancers, such as colorectal cancer (45.62%) and uterine cancer (46.94%) (unpublished data). We speculate that the erythropoietin-driven overproduction of RBCs stimulated by the pathogenesis of HCC partially solves the problem of oxygen-carriage by RBCs in the body, although the maturity of some erythroid cells in the peripheral blood of HCC patients is abolished.

Analysis of the RBC proteome is a major challenge in this field. Novel proteins have been identified, most of which are non-functionally characterized [44]. Submicron vesicles are derived from a parent cell and have different names depending on their size, ranging from 30–1000 nm, or on their production mode, including exocytosis, membrane budding, or apoptosis. These vesicles are called exosomes, microvesicles, microparticles, and so on. RBC vesicles contain RBC cytosol and are surrounded by RBC membrane, and derived by RBC during aging or after stimulation. In this study, we identified > 70% of proteins as components of RBC vesicles including intracellular and extracellular vesicles, of which > 90% are extracellular vesicle proteins that were also enriched in Fig 2B. The proteome profile of RBC vesicles during the tumorigenesis of hepatocellular carcinoma could provide new direction in the pathogenesis of HCC.

We revealed the presence of immature RBCs in the peripheral blood of patients with HCC. Physiologically, nucleated erythroid cells only exist in the bone marrow but not in peripheral blood in adults. The presence of these nucleated red blood cells in the peripheral blood may indicate a pathological status, including cancer. We speculate regarding the possible implications of the presence of immature erythroid cells in the peripheral blood of patients with HCC. First, the liver is an extramedullary hematopoietic organ, and CD71^+^CD45^+^ erythroid cells are characterized in situ in the tumor tissue of HCC [45]. These immature RBCs in situ-generated by tumor tissue are released into the peripheral blood. Second, under pathological conditions of HCC, the bone marrow may be stimulated to release nucleated erythroid cells into the peripheral blood. Peripheral RBCs may be a good indicator of tumor onset and development and may serve as a marker for the early diagnosis of cancers, including HCC.

Our study has some limitations. The sample size was small, especially for the evaluation of candidates using PRM, which may have caused the observed difference in the expression of some globins, such as HBE1 and HBA1, in proteomic data and the PRM evaluation. Moreover, our results provided the initial assessment for the early diagnosis of HCC with proteome profiles of RBCs from three groups, including HC, LC, and HCC. However, the sensitivity and specificity of candidates of interest in this application have not been evaluated in the current study. Furthermore, even if the method of isolating RBCs from peripheral blood is commonly used in laboratories, it may include non-RBC contaminants, such as leukocytes, which hinder the bioinformatic analysis. We identified some disrupted pathways, such as the autophagy pathway, which is also conserved in other blood cells. We speculate that the impurity of RBCs may explain this observation. This crude method will be adapted to further increase the accuracy of the detection. In addition, the autophagy pathway plays a role in the maturation of RBCs; during reticulocyte maturation, autophagy is responsible for the clearance of ribosomes and mitochondria to maximize the hemoglobin-carrying capacity of mature RBCs [46]. We identified the autophagy pathway during HCC tumorigenesis and speculate that another possibility is the defects in reticulocyte maturation during tumorigenesis of HCC. These reticulocytes are co-purified with mature RBCs during gradient centrifugation [46]. We found that the disrupted autophagy pathway in RBCs exists throughout the tumorigenesis of HCC, possibly representing the defects in reticulocyte maturation associated with the tumorigenesis of HCC. We propose that the autophagy pathway indicates the tumorigenesis of HCC.

## Conclusions

This study underlined the clinical relevance of proteins in RBCs and the tumorigenesis of HCC and identified candidate biomarkers for the early diagnosis of HCC from a new perspective. We revealed that molecular changes in RBCs are closely associated with HCC tumorigenesis. The defects in RBCs are initiated during LC and continue with the onset of established HCC, represented by the presence of immature erythroid cells. We discovered several novel biomarkers, including SMIM1 and ANXA7, in RBCs that could imply the early diagnosis of HCC. Our results provided a novel strategy that utilizes biochemical properties of RBCs for the early diagnosis of HCC, which may aid translational research and application in the diagnosis of HCC and may expand the clinical application of “liquid biopsies” in the early diagnosis of cancer.

## Supporting information

S1 Table Statistical analysis of clinical characteristics of the cohort. The sociodemographic and clinical characteristics are expressed as the mean ± SEM. ALT: alanine aminotransferase; AST: aspartate aminotransferase; NA: not available.
(DOCX)

S2 Table List of the total proteins identified by mass spectrometry in a cohort of LC, HCC, and HC patients. (XLSX)

S3 Table List of selected proteins verified in another cohort of patients with LC (n = 9), HCC (n = 11), and HC (n = 10) using an RPM strategy. SMIM1 and ANXA7 are the top two confirmed proteins.
(XLSX)

S4 Table List of PRM precursors qualified for quantification of the selected proteins. (XLSX)

S5 Table List of differentially expressed RBC proteins in patients with LC and healthy controls. (XLSX)

S6 Table List of differentially expressed RBC proteins in patients with HC and HCC. (XLSX)

S7 Table List of differentially expressed RBC proteins in patients with LC and HCC. (XLSX)

S1 File (RAR)
