# Supplementary material for: Proteome expression profiling of red blood cells during the tumorigenesis of hepatocellular carcinoma
Source: PLoS One. 2022 Nov 8;17(11):e0276904. doi: 10.1371/journal.pone.0276904 (PMC9642896; doi:10.1371/journal.pone.0276904)
Supplement: S1 Table — The sociodemographic and clinical characteristics are expressed as the mean ± SEM. ALT: alanine aminotransferase; AST: aspartate aminotransferase; NA: not available. (DOCX) [file pone.0276904.s001.docx]

**Table S1. Statistical analysis of clinical characteristics of the cohort**

| **Characteristics** | **HCC (n = 30)** | **LC (n = 17)** | **HC (n = 25)** |
| --- | --- | --- | --- |
| **Age (y)** | 55.77 ± 7.55 | 54.88 ± 15.54 | 55.08 ± 4.57 |
| **Gender** |  |  |  |
| Male | 26 | 13 | 17 |
| Female | 4 | 4 | 8 |
| **Stage at initial diagnosis** |  |  |  |
| I | 22 | NA | NA |
| II | 7 | NA | NA |
| III | 1 | NA | NA |
| **ALT (IU/L)** | 128.89±243.92*** | 25.79±16.00 | 23.75±13.97 |
| **AST (IU/L)** | 92.96±114.79*** | 35.48±18.59*** | 18.324±6.21 |
| **AFP (ng/mL)** | 4718.89 ± 15 511.58*** | 3.24 ± 2.17 | 2.6 ± 1.17 |
| **Hemoglobin (g/L)** | 131.87 ± 15.98** | 103.94 ± 19.26*** | 146.21 ± 16.12 |
| **Erythrocytes (10^12^/L)** | 4.19 ± 0.52*** | 3.55 ± 0.67*** | 4.76 ± 0.49 |
| **Leukocytes (10^9^/L)** | 8.34 ± 4.81 | 5.91 ± 7.09* | 5.71 ± 1.38 |
| **Platelets (10^9^/L)** | 127.47 ± 48.89*** | 105.30 ± 72.02*** | 192.84 ± 48.74 |

Note: Statistics were analyzed using the Wilcoxon rank-sum test. Significant differences between LC or HCC and HC were set as ***, *P* < 0.001; **, *P* < 0.01, *, *P* < 0.05.
